# Dementia Dialogue: An Educational Workshop for Medical Students

**DOI:** 10.1111/jgs.70096

**Published:** 2025-09-11

**Authors:** Kayla S. Murphy, Grant Flindt, Michelle Nguyen, Deborah Freeland

**Affiliations:** ^1^ Department of Internal Medicine University of Texas Southwestern Medical Center Dallas Texas USA; ^2^ University of Texas Southwestern Medical School, University of Texas Southwestern Medical Center Dallas Texas USA; ^3^ Division of Geriatric Medicine University of Texas Southwestern Medical Center Dallas Texas USA

**Keywords:** curriculum development, dementia, dementia education, medical student education

## Abstract

We created and hosted a dementia workshop for first and second year medical students in 2023 and 2024 aimed at increasing interest and passion for empathic dementia care. The workshop significantly increased students' comfort with dementia care topics and knowledge of dementia and delirium. We will continue this workshop annually with ongoing improvements based on student feedback.

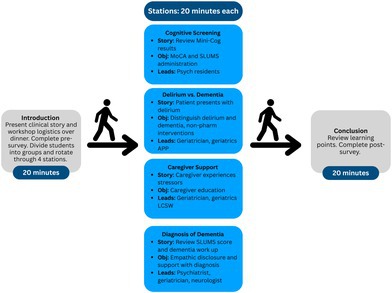

## 1 Introduction

As the incidence and prevalence of dementia continue to rise, physicians across specialties will likely provide care for individuals with dementia. The Geriatric Competencies for Graduating Medical Students emphasize that medical students should have an understanding of how to screen for and differentiate between dementia and delirium, as well as recognize non‐pharmacologic management of agitation [1]. Despite this, there are often limited opportunities to learn these skills, and prior research has shown that students may have negative attitudes toward caring for older adults with dementia [2]. Prior initiatives have focused on interdisciplinary dementia education for senior students [3]. To address these educational needs earlier in medical school, we created a workshop to provide pre‐clinical medical students with knowledge and tools for effective dementia care.

## 2 Methods

We developed a voluntary workshop for pre‐clinical medical students at the University of Texas Southwestern Medical School to increase knowledge and comfort in dementia care. We hosted the workshop in fall 2023 and 2024 during the neuroscience course. The workshop was advertised via flyer and championed by leaders of our Geriatric Medicine interest group and the course director. See Figure S1 for workshop objectives.

**FIGURE 1:**
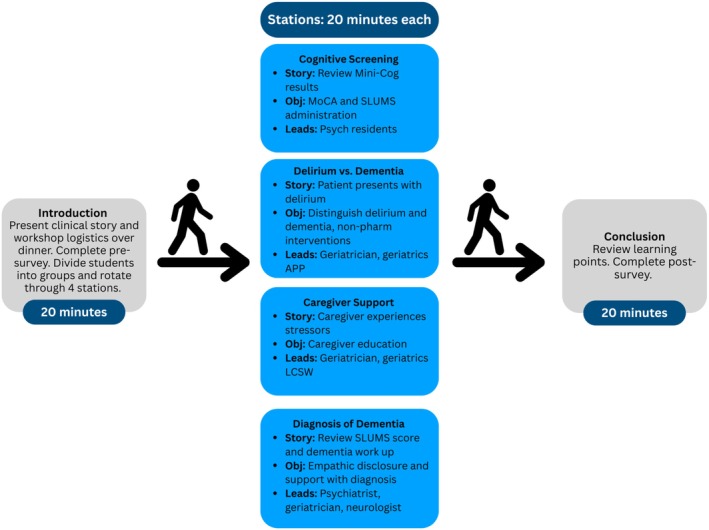
Structure of the fall 2024 Dementia Workshop. APP, Advanced Practice Provider; LCSW, Licensed Clinical Social Worker; MoCA, Montreal Cognitive Assessment; SLUMS, The Saint Louis University Mental Status test4 [4].

The workshop was facilitated on a weekday evening by a combination of psychiatry, neurology, and geriatric medicine faculty (physicians and nurse practitioners), as well as resident physicians and a licensed clinical social worker. Students had dinner, learned about workshop logistics, and received an introduction to the patient story. Students were divided into four groups and rotated through stations where additional aspects of the patient story were revealed and facilitators guided discussion with prompting questions (Figure S2); the four stations discussed cognitive screening, caregiver support, relaying a dementia diagnosis, and distinguishing dementia versus delirium with a focus on non‐pharmacologic behavioral management. The format of the fall 2024 workshop is detailed in Figure 1. Based on feedback from 2023, station duration was increased from 15 to 20 min.

Pre‐ and post‐workshop surveys were collected and included four 5‐point Likert scale questions to assess comfort with dementia care (1: “*very uncomfortable*,” 5: “*very comfortable*”), four free response questions to assess change in knowledge, and two free response questions to elicit feedback (Figure S3). The survey contained anonymous identifiers to match the pre‐ and post‐surveys.

We assessed differences for each student using a paired *t*‐test for continuous data and the McNemar's test for categorical data to account for within‐participant correlation. Data were checked for underlying assumptions such as normality. All statistical tests were conducted using a significance level of 0.05.

## 3 Results

Forty‐two first‐ and second‐year medical students participated in the fall 2023 workshop and 29 in the 2024 workshop. Of these, 42 of the pre‐ and post‐surveys were able to be matched and included in the analysis. The average Likert score increased significantly for students' comfort screening a patient for memory concerns (+1.5 ± 1.0, *p* < 0.001), sharing a diagnosis of dementia (+1.5 ± 0.9, *p* < 0.001), working with older adults with dementia (+1.0 ± 0.9, *p* < 0.001), and educating caregivers about dementia (+1.7 ± 1.0, *p* < 0.001) (Figure S4). On the post‐survey knowledge assessment, students had a higher percentage of correct answers when asked to distinguish between delirium and dementia (+47%, *p* < 0.0001), use screening tools for dementia (+38%, *p* < 0.05), and use screening tools for delirium (+40%, *p* < 0.0001). There was no significant difference between pre‐ and post‐survey knowledge of non‐pharmacologic interventions (+17%, *p* > 0.05) (Figure S5).

Across both workshops, 84% of students found the workshop helpful for developing their clinical skills. They appreciated small group interactions with clinicians and the relevant clinical pearls.

## 4 Discussion

This interprofessional and collaboratively led workshop offers a unique approach for students to learn about dementia through the lens of multiple specialties. Students' relative comfort with dementia care, including screening, discussing diagnoses, and providing caregiver support, increased significantly after the workshop. Knowledge of dementia and delirium also improved. Of note, students did not have significant improvement in their knowledge of non‐pharmacologic interventions for dementia. In this station, students were taught to distinguish between dementia and delirium and reviewed non‐pharmacologic interventions, so this station may need to be simplified. We suspect that fewer students attended the second iteration of the workshop because it was open to first‐ and second‐year students, some of whom may have attended the year prior. Ultimately, students found the workshop helpful for developing their clinical skills.

In an article published by Padala et al. [3], an interdisciplinary curriculum was created for fourth‐year medical students led by geriatricians and psychiatrists to address knowledge gaps in dementia care. Building on this, our workshop is a novel addition, because it includes multiple disciplines and specialties coming together to provide interactive dementia education during pre‐clinical training. This concept can be used to provide effective dementia education at other institutions and add practical skills and knowledge to the pre‐clinical medical school curriculum. The goal is to increase awareness, interest, and comfort navigating dementia care at an earlier stage in medical training.

We plan to continue this workshop annually with ongoing improvements. During the first two workshops, mild cognitive impairment was informally discussed in the cognitive screening and diagnosis of dementia stations, but we plan to ensure this is in the facilitator guide for future iterations. We seek to create opportunities for involvement of caregivers and/or persons with dementia with the inclusion of a formal assessment of student attitudes toward individuals with neurocognitive disorders.

Finally, our study had several limitations. Likert scales were used to assess students' relative comfort with workshop topics, but this is not a direct measure of clinical competency. Implementing an observed clinical scenario would allow for an objective competency assessment. The voluntary nature of our workshop may have also influenced the survey results. Additionally, not all institutions may be able to fund dinner, which contributed to student attendance. Continuing this initiative annually will allow for ongoing improvements as we aim to inspire interest and passion for empathic dementia care.

## Author Contributions

**Kayla S. Murphy:** participated in study design, acquisition of data, analysis of data, and preparation of the manuscript. **Grant Flindt:** participated in study design, acquisition of data, analysis of data, and preparation of the manuscript. **Michelle Nguyen:** participated in study design, acquisition of data, and preparation of the manuscript. **Deborah Freeland:** participated in study design, acquisition of data, and preparation of the manuscript.

## Disclosure

Sponsor's role: The Geriatric Medicine Division and Geriatric Psychiatry program at UT Southwestern sponsored this event by providing funding for food and advertising the event.

## Conflicts of Interest

The authors declare no conflicts of interest.

## Supporting information


**Data S1:** jgs70096‐sup‐0001‐Supinfo.pdf.
