# Supplementary material for: Dementia Dialogue: An Educational Workshop for Medical Students
Source: J Am Geriatr Soc. 2025 Sep 11;73(11):3636–8. doi: 10.1111/jgs.70096 (PMC12645555; doi:10.1111/jgs.70096)

Supplemental Figures:

Supplemental Figure S1. The five objectives of the dementia workshop for medical students.

**Workshop Objectives:**
1. By the end of this workshop, students will differentiate between delirium and dementia.
2. By the end of this workshop, students will demonstrate appropriate use of the tools necessary to screen for delirium and dementia.
3. By the end of this workshop, students will recognize the importance of educating patients and families about dementia and will feel more confident in using these skills.
4. By the end of this workshop, students will identify non-pharmacologic treatments for dementia.
5. By the end of this workshop, students will recognize strategies to best communicate with those experiencing dementia.

Supplemental Figure S2. Facilitator guide with longitudinal patient story.

Dementia Workshop Example Case:

Introduction:

Ms. DB is an 85 yo woman who comes to establish care with you in primary care clinic. Her daughter is present with her in the clinic room.

PMH: HTN, HLD, insomnia, migraines with aura, acid reflux, frequent falls, glaucoma, sensorineural hearing loss

You take the history with the daughter present, hearing from both Ms. B and her daughter.

In taking a social history, you find out that Ms. B is having some concerns about her memory, mostly that she is having trouble leading her Bible study and remembering names of people in the class. When her daughter adds history, like the fact that Ms. B forgot to pay her water bill a month ago and also that she has been burning food that she has left on the stove, Ms. B gets upset and defensive. She lives at home alone in a two-story house and was widowed in 2020.

Other history includes that she has been having trouble falling asleep, but also trouble staying asleep. She is independent in her activities of daily living (ADLs – toileting, bathing, dressing, walking, transferring, feeding). She still drives. She has been having trouble with her medications (some missed, sometimes blood pressure low concerning for taking too many), but she still does them independently. She has had a few falls recently. She fell coming down the stairs at her home, as well as once at church when she walked off the sidewalk to be able to walk past a few people.

You ask her daughter to step out during the physical exam, so you can also get a chance to speak to Ms. B alone and do cognitive testing. After the physical exam, you go to the waiting room to bring her daughter back, giving you the chance to speak to her daughter alone.

Additional Case Information for Cognitive Screening Station:

She went to college and also has a Master's Degree in Chemistry. She worked as a chemistry teacher. On her MiniCog, she scores 3/3 for delayed recall and her clock is drawn below:

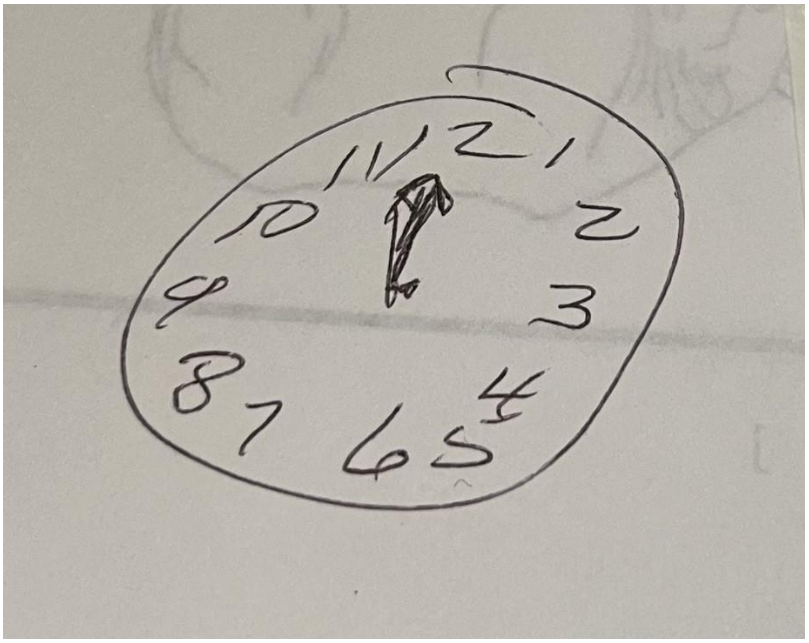

Review the steps of a MiniCog. What are your 3 words that you will use for your career? (Think of words with good category cues and ensure the words are not the first thing someone tends to guess in that category)

What cognitive testing will you do next?

What are the different domains tested by these additional tests?

Are there other things that look like dementia that you might want to assess for?

Practice one of the cognitive screening tests.

Additional Case Information for "Discussing a Diagnosis of Dementia" Station:

You are still at your first visit. Ms. B scores a 17/30 on the SLUMS.

-orientation: 3/3
-calculation: 0/3
-naming fluency: 3/3 (21 unique animals)
-short term recall: 3/5 (got one with category cue and one with multiple choice)
-attention: 0/2
-executive function: 2/4
-visuospatial: 2/2
-working memory: 4/8 (knew her name and what she did for work)
TOTAL: 17/30

She had recent blood work that shows normal TSH and vitamin B12 levels. Neurologic exam is non-focal. No known history of stroke. Based on history and testing, you are worried Ms. B has dementia.

How do you discuss the diagnosis?

How do you balance respecting your patients experiencing dementia and the need to obtain additional information from caregivers?

What emotions might you expect from someone receiving a diagnosis of dementia and how can you acknowledge these and support them?

Additional Case Information for "Caregiver Education" Station:

After your first visit with Ms. B, you asked her to allow her daughter to help her with medications and with bills. Her daughter calls your office a month later. She is very overwhelmed because her mother is upset at her when she tries to help with these instrumental activities of daily living. She also has a full-time job herself. They often fight because her mother repeats herself and never remembers what her daughter told her. The tension is palpable during the visits.

What can you suggest to help Ms. B's daughter as a primary caregiver for someone living with dementia?

What communication tools or strategies exist?

What resources exist?

Other scenarios:

Urinary incontinence starts. She doesn't want to wear the depends and the furniture is starting to smell like urine because she is having accidents.

Sleep remains an issue. She tends to sleep during the day, falling asleep in front of the TV. Then, she is up at night, often calling her daughter asking the same questions over and over.

Losing weight. She has lost 10 lbs in the last two months. She has not been trying to, and her BMI is 23.

Additional Case Information for "Dementia vs Delirium" Station:

In between visits, you get a phone call. Ms. B's daughter is very concerned about her mother, so you ask her to come in for an urgent appointment. Her daughter says Ms. B seems more confused than usual; she was not ready for her appointment, despite having reminded her the night before. She had trouble getting dressed (putting her shirt on backwards), which is not normally an issue. On medication reconciliation, you learn that she has started taking "Simply Sleep" which is an over-the-counter sleeping aid that contains diphenhydramine. She started this because she was still having trouble sleeping. On review of systems, you also find out that she is more constipated. On history, it is difficult to get details from Ms. B herself, as she keeps nodding off during the visit.

How can you differentiate between progression of dementia and delirium?

What is a way to screen for delirium?

How can you prevent or treat delirium?

A few years later, her daughter gets a job in LA. Ms. B moves into an assisted living facility to get more support. You get a call from the assisted living, saying that Ms. B is very resistant to taking showers. She hit someone trying to help her into the shower when they came up behind her and started trying to get her up. They are asking if you have a medication to prescribe for her agitation. She is often not wearing her hearing aids when you go to visit her.

What is going on here?

As most drugs used to treat behaviors related to dementia often work by sedation, what are non-pharmacologic ways to help Ms. B and her caregivers at the assisted living facility?

Supplemental Figure S3. Pre- and post-survey questions. A 5-point Likert scale was used, with 1 representing "very uncomfortable" and 5 representing "very comfortable." All knowledge-based questions were assessed by one evaluator who used a standard set of correct responses for grading. The post-workshop survey included two additional free response questions.

Pre-survey:

1. How comfortable do you feel screening a patient for memory concerns? (1 to 5 likert scale)
2. How comfortable do you feel sharing a diagnosis of dementia with a patient? (1 to 5 likert scale)
3. How comfortable do you feel working with older adults with dementia? (1 to 5 likert scale)
4. How comfortable do you feel educating caregivers on how to best support their loved ones with dementia? (1 to 5 likert scale)
5. Name one characteristic that differs between delirium and dementia.
6. Please list a screening tool used for dementia.
7. Please list a screening tool used for delirium.
8. Please list a non-pharmacologic treatment for dementia.

Post-survey:

1. How comfortable do you feel screening a patient for memory loss? (1 to 5 likert scale)
2. How comfortable do you feel sharing a diagnosis of dementia with a patient? (1 to 5 likert scale)
3. How comfortable do you feel working with older adults with dementia? (1 to 5 likert scale)
4. How comfortable do you feel educating caregivers on how to best support their loved ones with dementia? (1 to 5 likert scale)
5. Name one characteristic that differs between delirium and dementia.
6. Please list a screening tool used for dementia.
7. Please list a screening tool used for delirium.
8. Please list a non-pharmacologic treatment for dementia.
9. How helpful was this workshop in developing your clinical skills?
10. What would you change about this workshop?

Supplemental Figure S4. Likert scale responses from pre- and immediate post-surveys with the average change in Likert score between pre- and post-surveys included. Results from fall 2023 and fall 2024 workshops are combined.

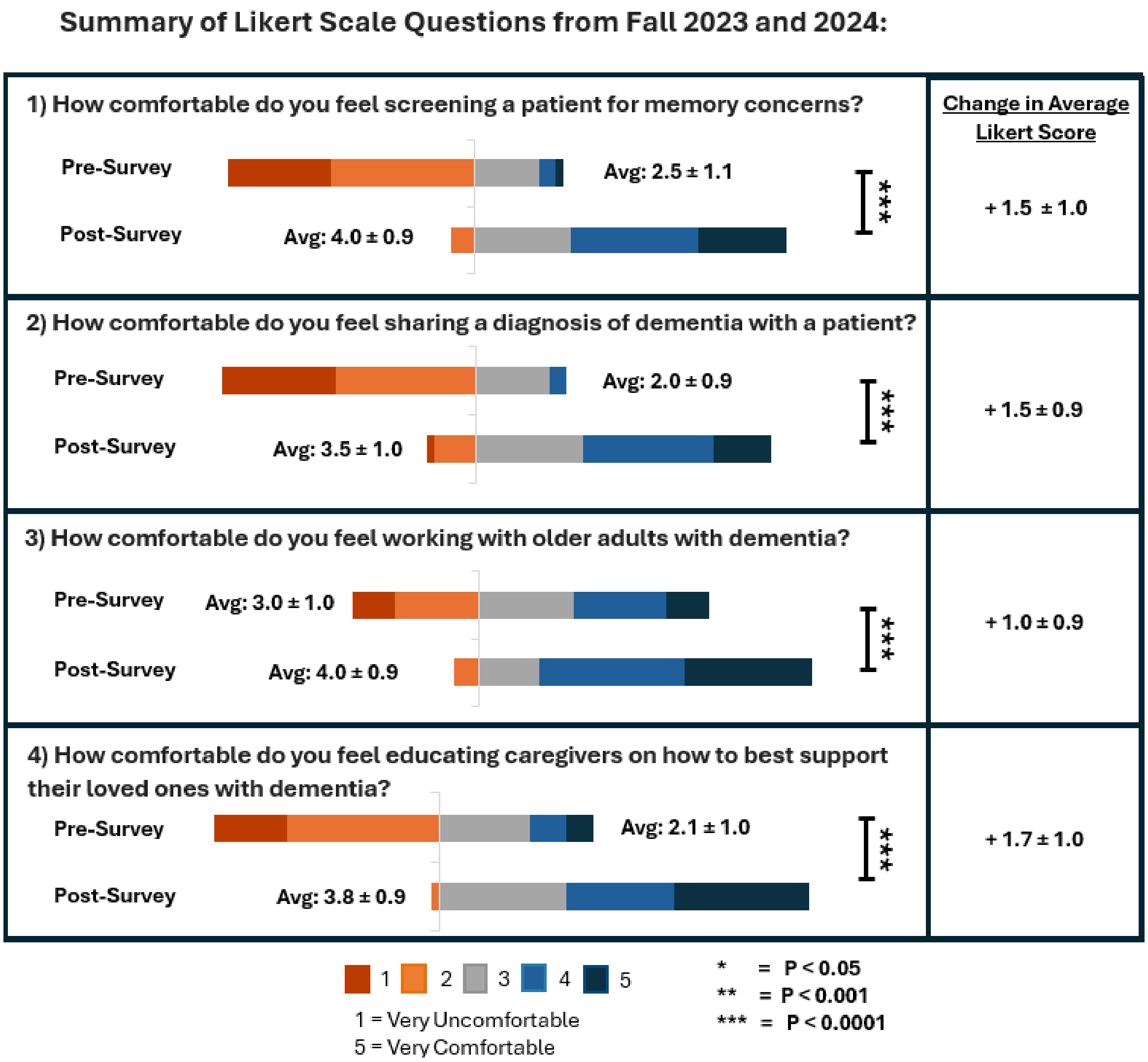

Supplemental Figure S5. Responses to knowledge-based questions before and immediately after the workshop. Results from fall 2023 and fall 2024 workshop are combined.

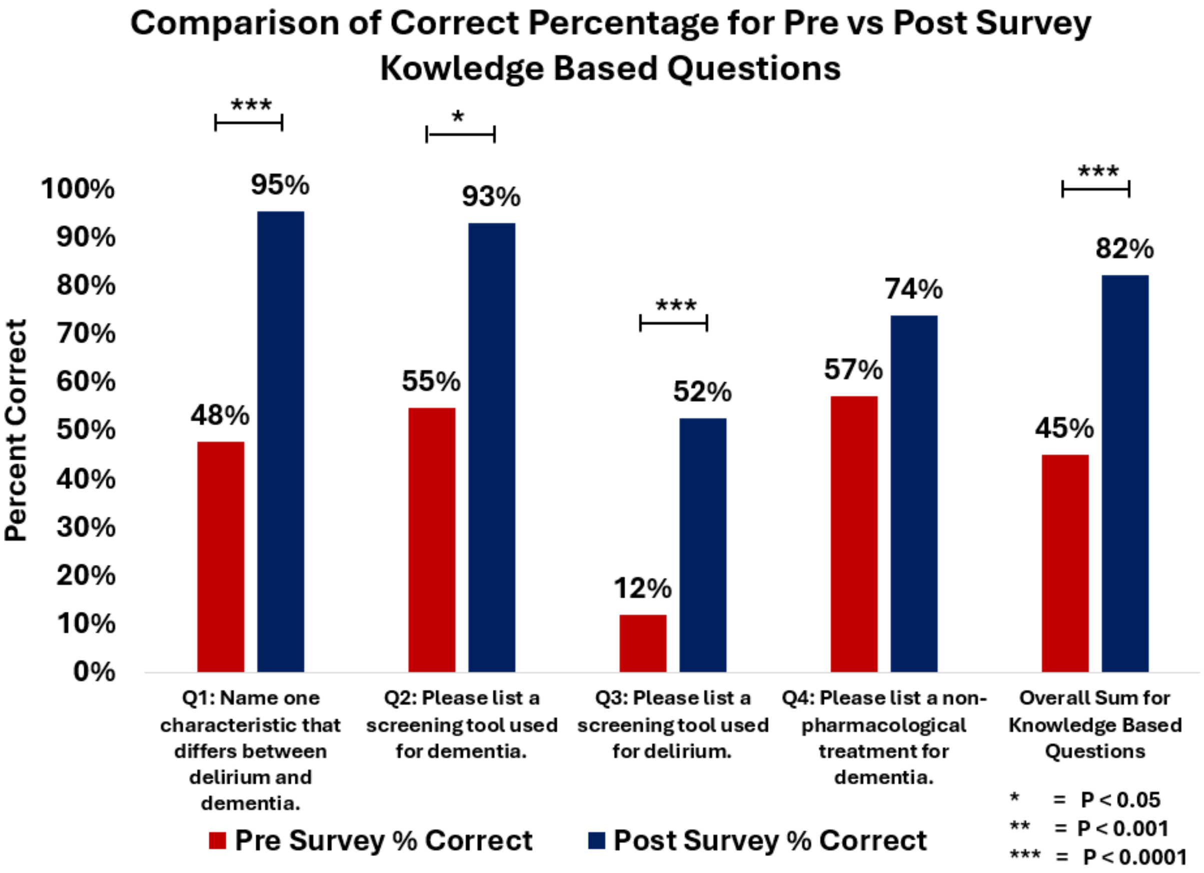

Supplement: Supplementary file 1 — Data S1: jgs70096‐sup‐0001‐Supinfo.pdf. [file JGS-73-3636-s001.pdf]
